# Mutational Profile of Metastatic Breast Cancers: A Retrospective Analysis

**DOI:** 10.1371/journal.pmed.1002201

**Published:** 2016-12-27

**Authors:** Celine Lefebvre, Thomas Bachelot, Thomas Filleron, Marion Pedrero, Mario Campone, Jean-Charles Soria, Christophe Massard, Christelle Lévy, Monica Arnedos, Magali Lacroix-Triki, Julie Garrabey, Yannick Boursin, Marc Deloger, Yu Fu, Frédéric Commo, Véronique Scott, Ludovic Lacroix, Maria Vittoria Dieci, Maud Kamal, Véronique Diéras, Anthony Gonçalves, Jean-Marc Ferrerro, Gilles Romieu, Laurence Vanlemmens, Marie-Ange Mouret Reynier, Jean-Christophe Théry, Fanny Le Du, Séverine Guiu, Florence Dalenc, Gilles Clapisson, Hervé Bonnefoi, Marta Jimenez, Christophe Le Tourneau, Fabrice André

**Affiliations:** 1 INSERM Unit U981, Gustave Roussy, Villejuif, France; 2 Department of Medical Oncology, Centre Léon Bérard, Inserm U1052, Lyon, France; 3 Biostatistics Department, Institut Claudius Regaud, IUCT-Oncopole, Toulouse, France; 4 Department of Medical Oncology, Institut de Cancérologie de l’Ouest, Nantes, France; 5 Department of Medical Oncology, Gustave Roussy, Villejuif, France; 6 Faculté de Médecine, Université Paris Sud, Kremlin-Bicêtre, France; 7 Drug Development Department (DITEP), Gustave Roussy, Villejuif, France; 8 Department of Medical Oncology, Centre François Baclesse, Caen, France; 9 R&D UNICANCER, Paris, France; 10 Bioinformatics core facility, Gustave Roussy, Villejuif, France; 11 Department of Medical Biology and Pathology, Translational research laboratory and biobank, Gustave Roussy, Villejuif, France; 12 Department of Surgery, Oncology and Gastroenterology, University of Padova, Padua, Italy; 13 Medical Oncology 2, Veneto Institute of Oncology IOV—IRCCS, Padua, Italy; 14 Department of Medical Oncology, Institut Curie, Paris & Saint-Cloud, France; 15 Department of Medical Oncology, Institut Paoli-Calmettes, Marseille, France; 16 Department of Clinical Research, Centre Antoine Lacassagne, Nice, France; 17 Department of Medical Oncology, Institut du Cancer de Montpellier, Montpellier, France; 18 Department of Medical Oncology, Centre Oscar Lambret, Lille, France; 19 Department of Medical Oncology, Centre Jean Perrin, Clermont-Ferrand, France; 20 Department of Medical Oncology, Centre Henri Becquerel, Rouen, France; 21 Department of Medical Oncology, Centre Eugène Marquis, Rennes, France; 22 Department of Medical Oncology, Centre Georges-François Leclerc, Dijon, France; 23 Department of Medical Oncology, Institut Claudius Regaud, IUCT-Oncopole, Toulouse, France; 24 UNICANCER Biobanking Center, Centre Léon Bérard, Lyon, France; 25 Department of Medical Oncology, Institut Bergonié, Université de Bordeaux, INSERM U916, Bordeaux, France; 26 EA7285, Versailles-Saint-Quentin-en-Yvelines University, Montigny-le-Bretonneux, France; Washington University School of Medicine, UNITED STATES

## Abstract

**Background:**

Major advances have been achieved in the characterization of early breast cancer (eBC) genomic profiles. Metastatic breast cancer (mBC) is associated with poor outcomes, yet limited information is available on the genomic profile of this disease. This study aims to decipher mutational profiles of mBC using next-generation sequencing.

**Methods and Findings:**

Whole-exome sequencing was performed on 216 tumor–blood pairs from mBC patients who underwent a biopsy in the context of the SAFIR01, SAFIR02, SHIVA, or Molecular Screening for Cancer Treatment Optimization (MOSCATO) prospective trials. Mutational profiles from 772 primary breast tumors from The Cancer Genome Atlas (TCGA) were used as a reference for comparing primary and mBC mutational profiles. Twelve genes (*TP53*, *PIK3CA*, *GATA3*, *ESR1*, *MAP3K1*, *CDH1*, *AKT1*, *MAP2K4*, *RB1*, *PTEN*, *CBFB*, and *CDKN2A*) were identified as significantly mutated in mBC (false discovery rate [FDR] < 0.1). Eight genes (*ESR1*, *FSIP2*, *FRAS1*, *OSBPL3*, *EDC4*, *PALB2*, *IGFN1*, and *AGRN*) were more frequently mutated in mBC as compared to eBC (FDR < 0.01). *ESR1* was identified both as a driver and as a metastatic gene (*n* = 22, odds ratio = 29, 95% CI [9–155], *p* = 1.2e-12) and also presented with focal amplification (*n* = 9) for a total of 31 mBCs with either *ESR1* mutation or amplification, including 27 hormone receptor positive (HR+) and HER2 negative (HER2−) mBCs (19%). HR+/HER2− mBC presented a high prevalence of mutations on genes located on the mechanistic target of rapamycin (mTOR) pathway (*TSC1* and *TSC2*) as compared to HR+/HER2− eBC (respectively 6% and 0.7%, *p* = 0.0004). Other actionable genes were more frequently mutated in HR+ mBC, including *ERBB4* (*n* = 8), *NOTCH3* (*n* = 7), and *ALK* (*n* = 7). Analysis of mutational signatures revealed a significant increase in APOBEC-mediated mutagenesis in HR+/HER2− metastatic tumors as compared to primary TCGA samples (*p* < 2e-16). The main limitations of this study include the absence of bone metastases and the size of the cohort, which might not have allowed the identification of rare mutations and their effect on survival.

**Conclusions:**

This work reports the results of the analysis of the first large-scale study on mutation profiles of mBC. This study revealed genomic alterations and mutational signatures involved in the resistance to therapies, including actionable mutations.

## Abstract 2

Fabrice Andre and colleagues describe the mutations occurring in a large group of patients with metastatic breast cancer.

## Author Summary

**Why Was This Study Done?:**

- Breast cancer often results in poor outcomes after it has metastasized to distant organs, but, while primary breast tumors have been extensively characterized at the molecular level, metastatic lesions are poorly understood.
- This study aims to characterize the mutational landscape of metastatic breast cancer by performing and analyzing whole-exome sequencing of a large collection of metastatic breast tumors and corresponding blood samples.
- Understanding of the mutational landscape of metastatic tumors should open new avenues for assessing resistance to therapy and developing better treatments.

**What Did the Researchers Do and Find?:**

- The authors generated a large collection of whole-exome sequencing data from the DNA of breast cancer metastases and from each patient’s corresponding unmutated DNA in order to identify mutations and gene copy number alterations specific to the tumors.
- The bioinformatics analyses identified recurrently mutated genes in metastatic tumors and revealed the genes specifically involved in metastatic disease by comparing their mutational frequency to those of primary breast tumors.
- The study allowed identification of the affected genes and of mutational signatures that were more prevalent in metastatic as compared with primary tumors and that may be involved in the resistance to therapies.

**What Do These Findings Mean?:**

- The identification of mutational and copy number alterations specifically involved in breast cancer metastasis demonstrated that tumors evolve under the pressure of therapy.
- Characterization of mutations and copy number alterations in metastatic lesions in addition to primary tumors should help to tailor treatment for patients, with the potential for improved clinical outcomes.

## Introduction

Major efforts have been made to characterize early breast cancer at the genomic level [1,2]. These efforts have led to extensive description of genomic alterations involved in tumorigenesis or tumor progression of early breast cancer. These studies report that early breast cancer includes a large number of rare segments characterized by actionable genomic alterations such as *PIK3CA* mutations, *ERBB2* amplification, *FGFR1* amplification, *CCND1* amplification, *AKT1* mutations, and *GATA3* mutations [1,2]. Follow-up studies report that C>T mutations at CpG sites are the major mutational pattern in early breast cancer [3]. Although sequencing of primary breast cancer has provided insight into the biology of early malignancy, around 80% of the patients presenting with such a disease will never relapse after conventional therapy. Therefore, understanding the biology of early breast cancer will not help in deciphering the specificities of the lethal disease or translate into treatment advances. Recent data from different types of cancer have suggested that there is a strong heterogeneity between primary tumors and metastases and that genomic profiles of metastases could dramatically differ from primary tumors. Gerlinger and colleagues have shown that only 30% of the mutations are shared between different tumor sites of kidney cancers [4]. Also, Haffner and colleagues have shown that lethal prostate cancer can derive from a minority subclone of the primary tumor [5]. There is therefore a need to extensively describe the genomic alterations observed in metastatic breast cancers in order to identify pathways involved in drug resistance and metastatic processes and to generate new strategies to treat these patients. To this end, we have performed whole-exome sequencing of 216 pairs of metastatic breast cancers and blood and report on the mutational landscape associated with lethal malignancy.

## Materials and Methods

The following methodology was specifically developed for this analysis and did not follow an established protocol or analysis plan.

### Patients

Metastatic breast cancer patients who underwent a biopsy in the context of the SAFIR01 [6] (NCT01414933), SAFIR02 (NCT02299999), SHIVA [7] (NCT01771458), and MOSCATO (NCT01566019) prospective trials were potentially eligible for this study. These French multicenter trials used high-throughput genome analysis on fresh frozen tumor biopsies as a therapeutic decision tool for metastatic cancer patients, with solid cancers (SHIVA and MOSCATO) or specifically with breast cancer (SAFIR01 and SAFIR02). SAFIR01 included patients with metastatic breast cancers resistant to therapy, and SHIVA and MOSCATO included patients with metastatic cancers eligible for phase I trial, while SAFIR02 included patients with metastatic breast cancers who were starting first- or second-line chemotherapy. Details of each trial are given in S1 Text. Exclusion criteria for the whole-exome sequencing analysis were defined as follows: small or no quantity of tumoral DNA, <30% cancer cells on the biopsy sample (from frozen tissue), and no blood sample available. With these criteria, we identified 86 tumor-normal pairs from patients included in the SAFIR01 trial, 80 pairs in the SAFIR02 trial, 35 pairs in the SHIVA trial, and 15 pairs in the MOSCATO trial (S1 Table). All patients gave their informed consent for translational research and genetic analyses of their somatic DNA. All the studies were approved by the relevant IRBs. Overall, whole-exome sequencing for a total of 216 pairs of metastatic tumor and unmutated DNA derived from corresponding blood samples was performed using Illumina technology. Estrogen (ER) and progesterone (PR) receptors were considered positive if >1% of the cancer cells were stained or when the case was reported positive in the case report form of the trial. HER2 status was determined locally.

### Statistical Consideration

Data were summarized by frequency and percentage for categorical variables and by median and range for continuous variables. Comparisons between groups were performed using the Mann-Whitney rank sum test for continuous variables and Chi square or Fisher's exact test for categorical variables. Overall survival (OS) was estimated by using the Kaplan-Meier method, and univariate analyses were performed using the log-rank test. OS was defined as the delay between the inclusion in the trial and death. Patients who were alive were censored at last follow-up news. The Cox proportional hazard regression model was used for multivariate analysis. All variables associated with *p* < 0.05 on univariate analysis were included in the model. All statistical tests were two sided, and differences were considered statistically significant when *p* < 0.05. Stata 13.0 software (StatCorp, College Station, Texas) or R version 3.2.2 were used for the statistical analyses. False discovery rate (FDR), used for correcting *p*-values for multiple hypothesis testing, was computed using the Benjamini-Hochberg procedure.

### Whole-Exome Sequencing

Genomic DNA was captured using Agilent in-solution enrichment methodology with their biotinylated oligonucleotides probes library (SureSelect All Exon V5, Agilent, or SureSelect Clinical Research Exome, Agilent), followed by 75-base paired-end massively parallel sequencing on Illumina HiSeq2500, HiSeq4000, or NextSeq500 (S2 Table). For detailed explanations of the process, we refer the reader to the publication by A. Gnirke and colleagues [8]. Sequence capture, enrichment, and elution were performed according to the manufacturer’s instruction and protocols (SureSelect, Agilent) without modification. Briefly, 600 ng of each genomic DNA was fragmented by sonication and purified to yield fragments of 150–200 bp. Paired-end adaptor oligonucleotides from Illumina were ligated on repaired, A-tailed fragments and then purified and enriched by 4–6 PCR cycles. Five hundred ng of these purified libraries was then hybridized to the SureSelect oligo probe capture library for 24 h. After hybridization, washing, and elution, the eluted fraction was PCR amplified for 10–12 cycles, purified, and quantified by qPCR to obtain sufficient DNA template for downstream applications. Each eluted-enriched DNA sample was then sequenced on an Illumina HiSeq2500/4000 or NextSeq500 as paired-end 75 b reads. Image analysis and base calling were performed using Illumina Real Time Analysis Pipeline version 1.12.4.2 with default parameters. Mean coverage was 83 +/− 18X for normal blood samples and 122 +/− 15X for tumor samples, with respectively 87% (77%–93%) and 90% (85%–95%) of the targeted regions covered at 20X or more (S2 Table).

### Somatic Mutation Calling

Fastq files were aligned to the reference genome hg19 with the BWA mem algorithm [9]. After alignment, the BAM files were treated for PCR duplicate removal and then sorted and indexed with Picard for further analyses. Base recalibration and local realignment around indels were done with GATK [10]. For defining somatic mutations, we used the Mutect [11] (version 1.1.7) algorithm for identifying substitutions and the Scalpel [12] algorithm (version 0.5.2) for identifying small insertions and deletions (indels). Indels occurring in regions with a high number of point mutations detected by Scalpel were filtered out using the GATK VariantFiltration tool with parameters set to 3 mutations in a window of 35 bp. We kept indels of a size lower than 35 bp. We then merged the output of Mutect and Scalpel and further filtered for mutations organized in a cluster of 3 mutations or more in a window of 35 bp using the GATK VariantFiltration tool. We defined the final list of somatic mutations with the following filters: frequency of the reads with the altered base in the tumor > 10%; number of reads with the altered base in the tumor sample ≥ 5; frequency of the reads with the altered base in the normal sample < 2%; number of reads with the altered base in the normal sample ≤ 3; and total coverage in normal and tumor samples ≥ 10. The resulting somatic mutations were annotated with the snpEff and snpSift algorithms [13], and we selected somatic mutations occurring in coding regions only. We removed variants that were also detected in at least one normal sample in our cohort or annotated as known polymorphisms (reported by 1000 Genomes or the ESP databases) unless the variant was also reported in Catalogue of Somatic Mutations in Cancer (COSMIC) [14] or ClinVar (http://www.ncbi.nlm.nih.gov/clinvar/). In order to control for possible biases due to the difference in bait territories from the two capture kits, we verified the mutations that were unique to one bait territory and found that 50 mutations involving 39 genes were unique to one of the bait but none of these mutations affected significantly mutated genes. We filtered six indels after manual inspection. We manually added 2 hotspot mutations (1 His1047Arg PIK3CA [COSM94986] and 1 Glu349* TP53 [COSM140784]) that were originally identified in the tumor in the clinical trial screenings and that were filtered by the somatic mutation filters because of the high frequency of the altered allele in the blood samples (respectively four supporting reads for an allele frequency of 0.022 and seven supporting reads for an allele frequency of 0.11), probably due to circulating tumor DNA. The list of mutations is reported in S3 Table. We computed the cancer cell fraction (CCF) of each mutation using the following steps: we first estimated the tumor purity with Sequenza [15] as well as the copy number at the mutated locus and the number of mutated alleles, as estimated by the altered reads allelic fraction [15]. We then computed the CCF of each mutation using the predicted tumor cellularity by Sequenza, the reference and variant allele read counts at the corresponding chromosomal position, and the estimated copy number at the locus following the framework previously proposed by Carter and colleagues [16]. Mutations were classified as clonal if the 95% confidence interval of the CCF overlapped 1 and as subclonal otherwise. To identify significantly mutated genes, we used the Mutation Significance (MutSig) [17], Mutational Significance in Cancer (MuSiC) [18], and Driver Genes and Pathways (drGAP) [19] algorithms. We defined significantly mutated genes as those with an FDR < 0.1 according to the MutSig algorithm that takes into account more parameters for identifying drivers than the other two algorithms, including gene size, background mutation rate, and replication timing.

### Copy Number Analysis

For deriving somatic copy number variations from whole-exome sequence data, we used the following strategy: we first computed the normalized ratio of reads between each tumor and corresponding normal sample using the package ExomeCNV in R and created the segmented profiles with the DNAcopy package. For defining amplifications and deletions, we used the Gistic2 algorithm [20] with the following thresholds for the log2 ratios: amp > 0.3 and del < −0.3. Gistic2 was run including all the samples and specifically for the HR+/HER2− samples and for the HR−/HER2− samples in order to control for disease subtypes. Focal peaks are listed in S4 Table.

### Mutational Processes

De novo mutational signature analysis was done using the Matlab Welcome Trust Sanger Institute’s signature framework. We used the deconstructSigs R package [21] to determine the contribution of the known signatures that explain each sample mutational profile with more than 50 somatic mutations. We considered the 13 signatures (Signatures 1, 2, 3, 5, 6, 8, 10, 13, 17, 18, 20, 26, and 30) operative in breast cancer as defined in COSMIC (http://cancer.sanger.ac.uk/signatures/matrix.png). A signature was defined as operative or predominant if its contribution to the mutational pattern was respectively >25% (or >100 mutations) or >50%.

### The Cancer Genome Atlas (TCGA) Data

Somatic mutations for breast cancer TCGA cohort were extracted from the genome.wustl.edu_BRCA.IlluminaGA_DNASeq.Level_2.5.3.0.somatic.maf file available for download on the TCGA data matrix website, with somatic mutations available for primary tumors of 772 patients. We extracted ER, PR, and HER2 status from the clinical file downloaded from the TCGA data matrix, retrieving 419 HR+/HER2−, 100 HR−/HER2−, and 145 HER2+. In order to fairly compare mutational loads between TCGA and the metastatic cohort, we downloaded raw data for 33 randomly selected TCGA patients and processed the BAM files with the same pipeline described in this manuscript. We found that the number of mutations identified by our pipeline and by the TCGA pipeline was very similar (S1 Fig, linear regression R^2^ = 0.98, *p* < 2e-16). We also verified the identity of mutations called by the two pipelines and found that 80% of the mutations were common to the two pipelines (S2 Fig). Therefore, we used the somatic mutations as defined in the TCGA maf file for comparing the mutation frequencies of the genes.

## Results

### Patient Characteristics

The population analyzed in the current study included 216 pairs of tumor and normal blood DNA from patients with metastatic breast cancer. Patients were classified in three subgroups according to hormone receptors (HRs; estrogen and progesterone receptors) and HER2 status (Table 1). One hundred and forty-three patients (66%) presented with HR+/HER2− breast cancer, 51 (24%) with triple-negative breast cancer, and 14 (6%) with HER2-overexpressing breast cancer. Ninety-four percent of the patients had received prior chemotherapy, and 120 (84%) of the patients with HR+/HER2− disease had received prior endocrine therapy.

**Table 1:** Patient characteristics.

	Overall (*n* = 216)	HR+/HER2− (*n* = 143)	HR−/HER2− (*n* = 51)	HER2+ (*n* = 14)	*p*-Value
**Age at Inclusion**					*p* = 0.0275
Median	54	55	48	51	
(Range)	(26–82)	(26–82)	(29–76)	(37–73)	
**Number of Metastatic Sites**					*p* = 0.5331
1–2	123 (57.2%)	80 (55.9%)	29 (58.0%)	10 (71.4%)	
>2	92 (42.8%)	63 (44.1%)	21 (42.0%)	4 (28.6%)	
Missing	1	0	1	0	
**Previous Endocrine Therapy**					*p* < 0.0001
No	73 (33.8%)	23 (16.1%)	43 (84.3%)	7 (50.0%)	
Yes	143 (66.2%)	120 (83.9%)	8 (15.7%)	7 (50.0%)	
Missing	0	0	0	0	
**Interval Metastatic Relapse/Inclusion (months)**					*p* < 0.0001
Median	8.3	15.5	1.2	0.8	
(Range)	(0.0–177.2)	(0.0–177.2)	(0.1–35.7)	(0.1–53.0)	
Missing	7	4	1	2	

### Genes Mutated in Metastatic Breast Cancers

**Fig 1:**
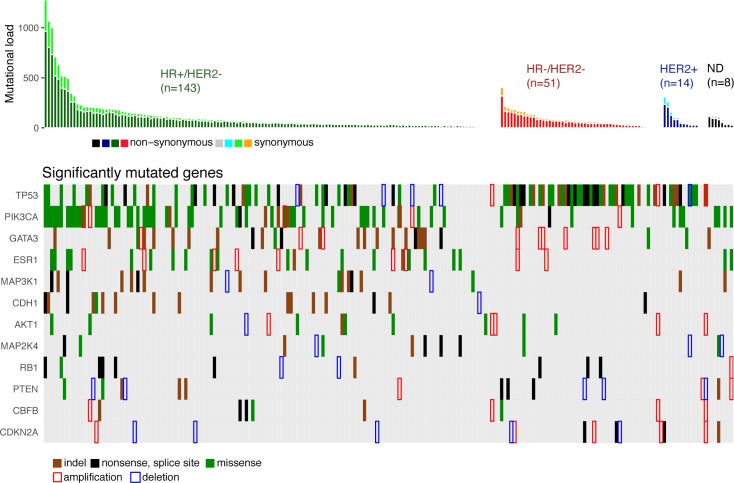
Driver gene mutations in metastatic breast cancers. The top panel shows the synonymous and nonsynonymous mutation rates (number of mutations) per patient according to the molecular subtype of the metastasis. HR, hormone receptor; ND, not determined. The bottom panel shows the significantly mutated genes according to MutSig analysis at FDR < 0.1. Amplifications and deletions correspond to the thresholded values from the Gistic2 output (respectively +2 and −2 values).

We identified 12 driver genes using the MutSig algorithm (FDR < 0.1) (Fig 1, S5 Table). Ten of these genes (*TP53*, *PIK3CA*, *GATA3*, *MAP3K1*, *CDH1*, *AKT1*, *MAP2K4*, *PTEN*, *CBFB*, and *CDKN2A)* have been previously shown to be frequently mutated in primary breast cancers (>2%, TCGA). In particular, *TP53* was mutated in 27% of HR+/HER2− metastatic breast cancer (mBC) as compared to 20% in HR+/HER2− early breast cancer (eBC) (Fisher Exact Test *p* = 0.13) while *PIK3CA* was mutated in 37% of the HR+/HER2− mBC and in 40% in eBC.

Two of the driver genes observed in mBC (*ESR1* and *RB1*) were infrequently mutated in primary tumors (<1% of HR+/HER2− eBC [TCGA]). Twenty-four mutations of *ESR1* were identified (1 synonymous, 2 indels, and 21 missense mutations) for a total of 22 mBCs, and these included 22 mutations in 20 out of 143 HR+/HER2- mBCs (14%). All ESR1 mutations occurred in the hormone receptor domain (S3 Fig) and included mutations in previously reported hotspots [22–24], as well as 2 new mutations (S3 Table). All of these 22 patients had received prior endocrine therapy. *RB1* was mutated in 7 out of 143 HR+/HER2− mBCs (5%) and 3 out of 51 HR−/HER2− mBCs (6%). Most of the mutations were disruptive, leading to truncated proteins (5 nonsense mutations, 3 splice sites, 1 indel, and 2 missense mutations, S4 Fig). When considering the estimation of the percentage of tumor cells harboring the mutation, i.e., CCF, we found that ESR1 and RB1 mutations were mostly identified as subclonal (ESR1: 14/21 mutations [67%]; RB1: 5/10 mutations [50%]). In comparison, PIK3CA and TP53 mutations were identified as subclonal for respectively 32% and 37% of their nonsynonymous mutations.

### Mutations Enriched in mBCs

**Fig 2:**
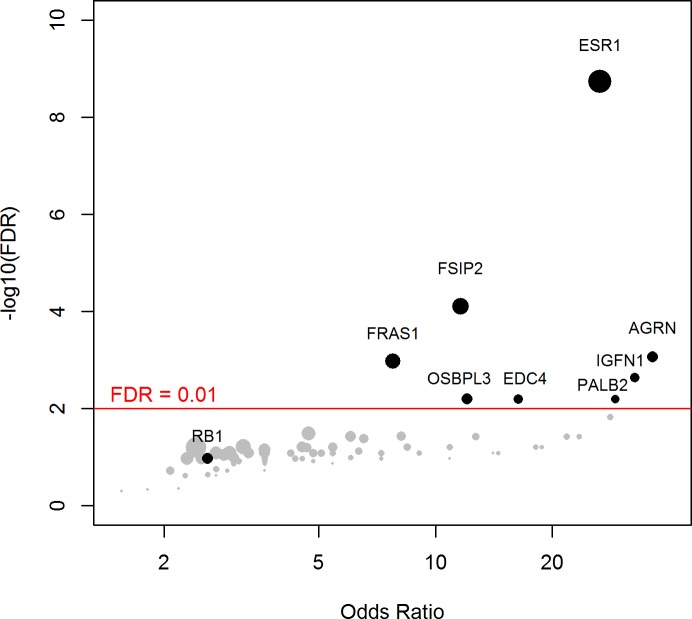
Genes more frequently mutated in mBC as compared to eBC (TCGA). The axes show the odds ratio calculated as the ratio of gene frequencies (*x*-axis) and the −log10 of the FDR of a Fisher exact test (*y*-axis) comparing the gene frequencies in metastatic versus primary tumors. The size of the points is proportional to the mutation frequency of the gene in the metastatic cohort. Highlighted points correspond to FDR < 0.01 or to significantly mutated genes.

**Fig 3:**
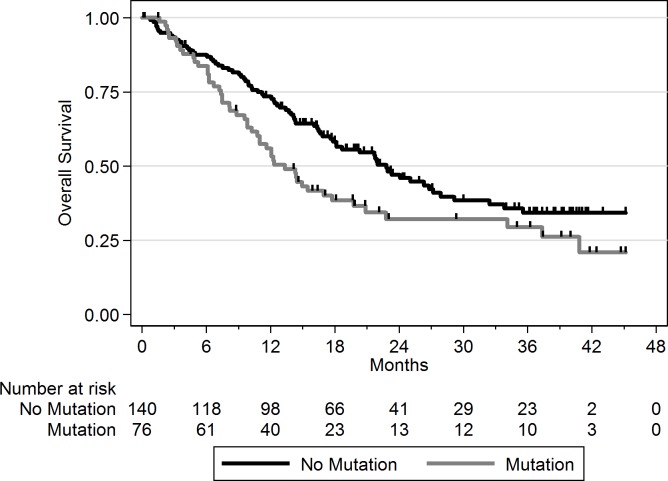
OS according to the presence of a mutation in one of the eight genes enriched in mBC as compared to eBC at FDR < 0.01. No mutation = mBC patients with tumors with no somatic mutation in the eight genes; mutation = mBC patients with tumors carrying at least one somatic mutation in the eight genes.

Using a FDR < 0.1, 199 genes out of 1,569 genes tested were more frequently mutated in mBC (*n* = 216) as compared to eBC (TCGA) (S6 Table). When a FDR < 0.01 was applied, 8 genes (*ESR1*, *FSIP2*, *AGRN*, *FRAS1*, *IGFN1*, *EDC4*, *OSBPL3*, and *PALB2*) were found to be more frequently mutated in mBC as compared to eBC (Fig 2). None of these genes, except *ESR1*, were identified as a driver using MutSig. However, *OSBPL3* and *PALB2* were both identified as drivers by MuSiC and drGAP at an FDR < 0.1 (S5 Table). *PALB2* was mutated in eight (4%) samples, while only one (0.1%) eBC was mutated in TCGA (FDR for enrichment in mBC = 0.006). Out of the 8 *PALB2* mutations, 5 were found in HR+/HER2− mBC. None of the cases with *PALB2* somatic mutations presented with a *PALB2* deleterious germline polymorphism in the other allele. We analyzed outcome data for comparing the OS of patients with metastatic tumors carrying at least one of the mutations enriched in the metastatic setting (*n* = 76) to the rest of the population (*n* = 140). Results of the univariate and multivariate analyses are reported in S7 and S8 Tables. In a multivariate analysis, mBC with at least one mutation in the 8 genes enriched in the metastatic setting presented a 2-fold increase in the hazard of death (hazard ratio = 1.97, 95% CI: 1.34–2.89, *p* = 0.001). Survival curves are reported in Fig 3.

**Fig 4:**
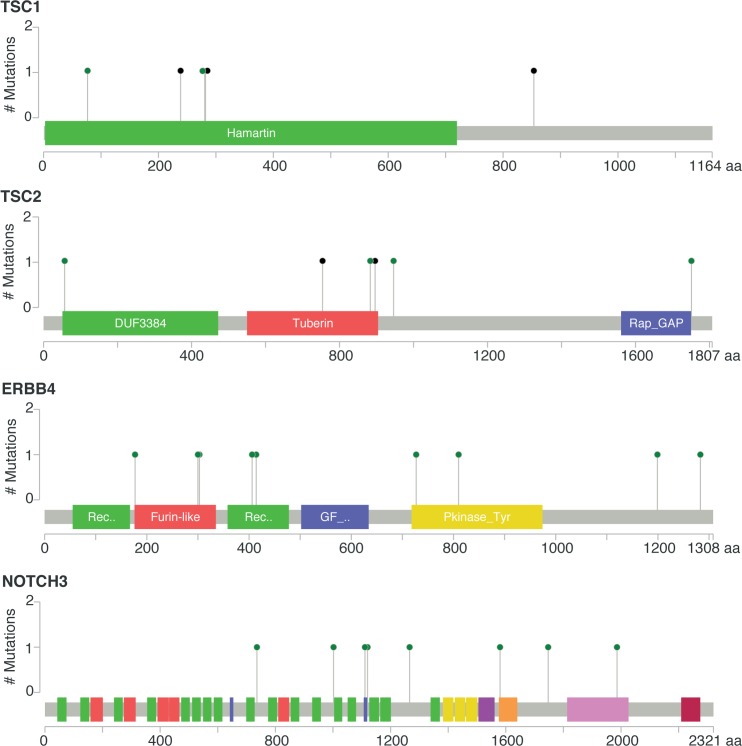
Somatic mutations of genes *TSC1*, *TSC2*, *ERBB4*, and *NOTCH3* in mBC (from cBioPortal). Green dots represent missense mutations, while black dots represent truncating mutations.

As this analysis might be biased by the difference in distribution of HR and HER2 subtypes between eBC and mBC, we also focused the analysis on the HR+/HER2− subtype (*n* = 143), in which 278 genes were more frequently mutated in mBC as compared to eBC (FDR < 0.1, S6 Table). Several of these genes were considered actionable. *TSC1* and *TSC2* were mutated in five (3.5%) and four (2.8%) samples, respectively (Fig 4). Overall, 6.3% of HR+/HER2− mBC presented an alteration in TSC1/2 as opposed to 0.7% of HR+/HER2− eBC (TCGA, *p* = 0.0004). Other actionable genes were more frequently mutated in HR+ mBC with an FDR < 0.1. These include *ERBB4* (nine missense mutations, including the two mutations COSM4764538 and COSM1015992, involving eight mBCs [five HR+/HER2−]), *NOTCH3* (eight missense and one splice site mutation(s) involving seven mBCs [four HR+/HER2−]), *ALK* (five missense and two splice site mutations in seven mBCs [six HR+/HER2−]), *EZH2* (two missense and one splice site mutation(s), including COSM220530, involving three HR+/HER2− mBCs) and *BRAF* (four missense mutations, including one COSM476, involving four mBCs [three HR+/HER2−]). The consequence of these mutations on the activity of the encoded proteins was difficult to assess as, even though ERBB4 and NOTCH3 mutations were all missense mutations (except for one splice site mutation in NOTCH3), they were located in different protein domains with no apparent hotspot (Fig 4).

### Mutational Signatures in mBCs

**Fig 5:**
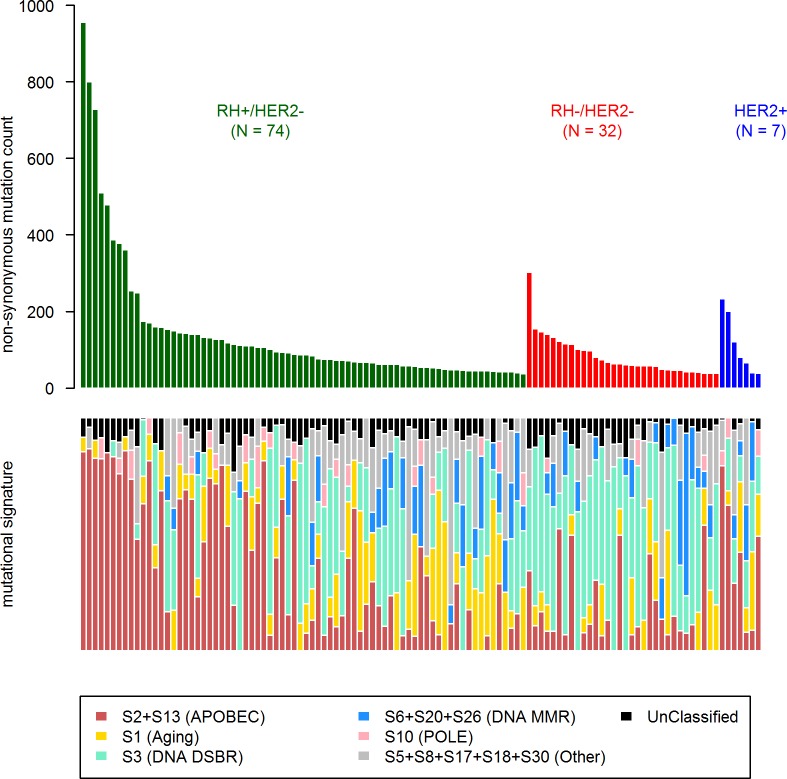
COSMIC mutational signature contribution in mBC. DNA DSBR, DNA double-strand break-repair by homologous recombination; DNA MMR, DNA mismatch repair.

**Fig 6:**
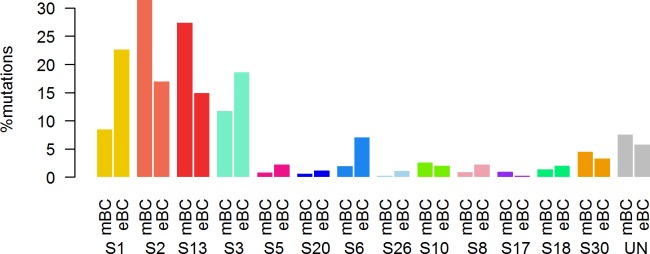
Distribution of the number of mutations according to mutational signatures in HR+/HER2− metastatic and primary (TCGA) breast tumors.

First, in order to identify a potential metastatic-specific mutational signature, we performed de novo mutational signature analysis that revealed five signatures operative in metastatic and primary breast cancer [25], but none of these signatures were specific to the metastatic setting (S1 Text, S9 Table and S5 and S6 Figs). We then assessed the contribution of 13 mutational signatures [25] in 118 metastatic samples and 278 primary tumors from TCGA presenting >50 mutations (S10 Table). Among the 13 signatures previously identified as operative in primary breast cancer, the most represented signatures in the metastatic samples were signature 1, related to aging; signatures 2 and 13, related to APOBEC3B activity; signature 3, associated with failure of DNA double-strand break-repair by homologous recombination; and signature 6, associated with defective DNA mismatch repair (Fig 5). While the identity of the signatures remained the same between primary and metastatic samples, their contribution dramatically changed, especially in the HR+/HER2− subtype (Fig 6 and S7 and S8 Figs). Of note, the signatures related to the APOBEC3B enzyme (signatures 2 and 13) contributed to 58.8% of the mutations of the HR+/HER2− metastatic tumors as compared to only 31.9% in the primary TCGA samples (*p* < 2e-16), confirming previous work demonstrating a link between APOBEC-mediated mutagenesis and the acquisition of subclonal mutations [26].

### Copy Number Alterations in mBCs

Gistic2 analysis using sequence-based levels reported regions previously described to drive oncogenesis of primary tumors including amplified genes *CCND1*, *ERBB2*, and *MYC* and lost gene *PTEN* (S4 Table). In addition to these previously reported gene amplifications and deletions, the current study identifies a focal amplification of the *ESR1* locus confirming the mutational pattern of the gene. *ESR1* was amplified in 7 HR+/HER2− mBCs for a total of 27 HR+/HER2− mBCs (19%) with either *ESR1* mutation or amplification (Fig 1). Additionally, *RB1* was lost in 2 HR+/HER2− mBCs for a total of 9 samples (6%) with either RB1 mutation or homozygous deletion.

Finally, we computed two indices to describe the chromosomal instability of the metastatic samples based on the copy number analysis as previously described [27]: a global genomics index (GGI) and the number of breakpoints per sample (S1 Table). We found that the number of mutations per tumor did not correlate with either the GGI or the number of break points (S9 Fig). We also verified that the mutational load and the chromosomal instability were not affected by the tumor cell content of the samples. While we found that there was no correlation between cellularity and estimated chromosomal instability, we found a positive correlation between the percentage of tumor cells and the number of mutations (Pearson’s cor = 0.16, *p* = 0.02). However, among the five samples with no nonsynonymous mutations, only two samples had <50% tumor cells, while the other three had >70% tumor cells.

## Discussion

In the present manuscript, we have described the mutational landscape of 216 mBCs. This study reported genes significantly mutated in mBC and genes significantly more mutated in mBC as compared to eBC. HR+/HER2− mBC presented the most differences with HR+/HER2− eBC, including an increased mutational signature linked to APOBEC3B activity and a higher prevalence of actionable genes that may represent new strategies for mBC treatment.

Using a stringent definition (MutSig, FDR < 0.1), the current study identified *ESR1* and *RB1* as driver genes that are specific to mBCs. Previous studies [23,24,28] have already reported that ESR1 mutations could be acquired during the disease evolution and could mediate resistance to endocrine therapy. In the present study, we confirm that mutation of ESR1 is the most frequent “metastasis-specific” mutation observed in mBC. As expected, all the 22 patients who presented ESR1 mutations were ER+ and resistant to endocrine therapy. ESR1-mutated mBC could be a genomic segment defining an unmet medical need, for which fast-track approval of new agents is required. Rb1 is a tumor suppressor protein involved in cell cycle and phosphorylated by CDK4. The protein is required for the bioactivity of palbociclib (CDK4 inhibitor), a drug recently approved to treat HR+/HER2− mBC [29]. The present study identifies *RB1* mutations, most of them pointing to a loss of function of the protein, as driver alterations in mBCs; while this gene is almost never mutated in HR+/HER2− eBC (<1%), it was found mutated in 5% of the HR+/HER2− mBC (*p* = 0.008, FDR = 0.09). This finding suggests that a subset of HR+/HER2− mBC is deficient for RB1 and could present a primary resistance to CDK4 inhibitors. If validated, this finding suggests that *RB1* mutations should be assessed on metastatic samples before starting CDK4 inhibitors.

Several genes were more frequently mutated in mBC as opposed to eBC but did not meet the criteria for drivers using the MutSig algorithm. *PALB2* is a partner of BRCA1/2 and is involved in Fanconi anemia. Heterozygous loss-of-function mutations in *PALB2* have been shown to be a risk factor for breast cancer, while *PALB2* germline mutations have recently been associated with a poor outcome [30]. Several studies have suggested that PALB2-deficient cancers could be sensitive to PARP inhibitors. In the present study, *PALB2* somatic mutations were found in 4% of metastatic samples (*n* = 8), while the gene is mutated in only 0.1% of eBC (FDR = 0.006). The present results suggest that there is a population of PALB2-deficient mBC in which PARP inhibitors could be evaluated. Genes located on the mTOR pathway (*TSC1* and *TSC2*) were more frequently mutated in HR+/HER2− mBC (6%) as opposed to HR+/HER2− eBC (0.7%). All these mutations were observed in patients previously treated with endocrine therapy, suggesting that it could be a mechanism of resistance. mTOR inhibitors (everolimus) have been approved in HR+/HER2− mBC [31]. While this drug prolongs progression-free survival (PFS) for a majority of patients, only a few percent of them are outlier responders to this drug, and there is currently no molecular alteration that explains such cases. Further studies should evaluate whether the subset of patients with genomic alterations on mTOR pathways (*TSC1* and *TSC2*) could be outlier responders to everolimus. Other actionable genes were more frequently mutated in HR+ mBC, including *ERBB4*, *NOTCH3*, and *ALK*.

Analysis of mutational processes did not identify any signature specific to the metastatic setting but revealed a high increase in APOBEC-mediated mutations in HR+/HER2− mBC as compared to eBC. As for metastatic-specific mutations identified, this might also present a mechanism of resistance to therapy that needs further careful investigation.

The study included 216 sample pairs classified in three different classes based on hormonal receptor expression in the tumors, the largest group being HR+/HER2− (*n* = 143). Although this is the largest effort for profiling the mutational landscape of mBC to this day, the size of the cohort presents a limitation to the identification of rare events, especially in the triple-negative and HER2+ groups. Our ability to provide an exhaustive picture of mutational profiles of mBC may be limited by two main biases. The first bias comes from the absence of bone metastases in the study, due to the difficulty of extracting DNA from these lesions. A second potential bias may come from our inability to identify those mutations leading to a disease so aggressive that the patients will not be eligible for trial recruitment, preventing an exhaustive picture of the mutational profiles of mBC. However, recent studies on first-line therapies for advanced breast cancer have shown that early death is limited [32,33], and it is therefore unlikely that it has dramatically impacted the analysis. Additionally, there is a chance that mutations enriched in metastasis might be subclonal driver events and therefore might not be such good drug targets. The comparison of gene mutational prevalence between mBC and eBC suffers from two limitations. First, it would have been ideal to directly compare the mBCs with their corresponding primary tumor profiles, but this was not possible because of the obvious reason of sample availability. Second, we used mutational profiles of TCGA tumors that were identified by the TCGA team, whereas it would have been ideal to run the pipeline used for mBC on the TCGA data. Although we controlled for major biases, the use of different bioinformatics pipelines may have some unexpected consequences. It should also be noted that the copy number analysis is limited by the nature of the sequencing data, which does not allow for a uniform coverage of the genome. Finally, the survival analysis based on the mutational status of the eight metastasis-specific genes was independent from any other parameters such as mutational load or mutational signature contribution, making it difficult to establish a causal link between mutated genes and prognosis.

The dataset and the accompanying analysis described in this study provide a better understanding of the genetic basis of mBC and how much it differs from that of primary breast tumors. This study demonstrated that profiling metastatic cancer can be a major step in defining optimal treatments for patients, as new mutation events and processes may arise during cancer treatment. Follow-up studies will be essential for validating resistance mechanisms identified in this study.

## Supporting Information

S1 Fig Comparison of the number of somatic mutations per TCGA breast cancer case as identified by the TCGA pipeline and the pipeline developed by the Gustave Roussy (GR) team presented in detail in the Materials and Methods section. (PDF)

S2 Fig Comparison of the identity of the somatic mutations identified by the TCGA pipeline and the pipeline (GR) presented in detail in the Materials and Methods section. (PDF)

S3 Fig ESR1 somatic mutations. Green dots represent missense mutations, while brown dots represent indels.
(PDF)

S4 Fig RB1 somatic mutations. Green dots represent missense mutations, while black dots represent truncating mutations.
(PDF)

S5 Fig Profiles of the two mutational signatures obtained after de novo mutational signature analysis with metastatic tumor samples. (PDF)

S6 Fig Profiles of the five mutational signatures obtained after de novo mutational signature analysis with metastatic and primary tumor samples. (PDF)

S7 Fig Percentage of mutations according to mutational signature in HR−/HER2− metastatic (mBC) and primary (eBC) breast tumors. (PDF)

S8 Fig Percentage of mutations according to mutational signature in HER2+ metastatic (mBC) and primary (eBC) breast tumors. (PDF)

S9 Fig Correlation between number of somatic mutations and chromosomal instability or tumor cell content. (PDF)

S1 Table Clinical and molecular information for the 216 patients included in the study (XLSX)

S2 Table Quality controls for 432 whole exomes. (XLS)

S3 Table List of somatic mutations. (XLSX)

S4 Table Gistic2 results. (XLSX)

S5 Table FDR for significantly mutated genes by the three algorithms MutSig, MuSiC, and drGAP. (XLSX)

S6 Table FDR for enrichment of mutated genes in metastasis as compared to primary tumors. (XLSX)

S7 Table Univariate analysis for OS. (DOCX)

S8 Table Multivariate analysis for OS. (DOCX)

S9 Table De novo mutational signature analysis; percentage of eBC and mBC tumors with signature operative. (XLSX)

S10 Table Mutational signature contribution (number of mutations and percentage of mutations). (XLSX)

S1 Text Supporting text. (DOCX)

S2 Text Strengthening the Reporting of Observational Studies in Epidemiology (STROBE) checklist. (DOC)

## Abbreviations

CCF: cancer cell fraction
COSMIC: Catalogue of Somatic Mutations in Cancer
drGAP: Driver Genes and Pathways
eBC: early breast cancer
FDR: false discovery rate
GGI: global genomics index
HR: hormone receptor
mBC: metastatic breast cancer
MOSCATO: Molecular Screening for Cancer Treatment Optimization
mTOR: mechanistic target of rapamycin
MuSiC: Mutational Significance in Cancer
MutSig: Mutation Significance
OS: overall survival
PFS: progression-free survival
STROBE: Strengthening the Reporting of Observational Studies in Epidemiology
TCGA: The Cancer Genome Atlas
