# Supplementary figures and images for: Mutational Profile of Metastatic Breast Cancers: A Retrospective Analysis
Source: PLoS Med. 2016 Dec 27;13(12):e1002201. doi: 10.1371/journal.pmed.1002201 (PMC5189935; doi:10.1371/journal.pmed.1002201)

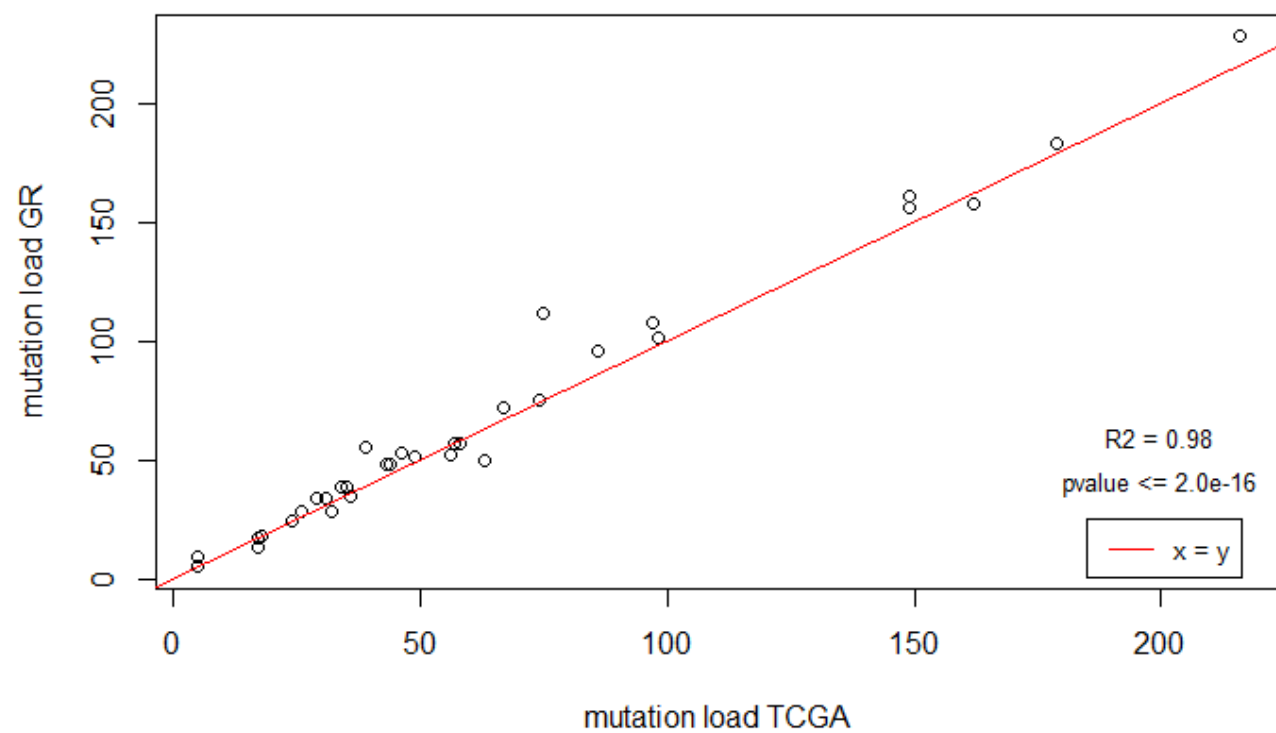

Supplement: S1 Fig — (PDF) [file pmed.1002201.s001.pdf]

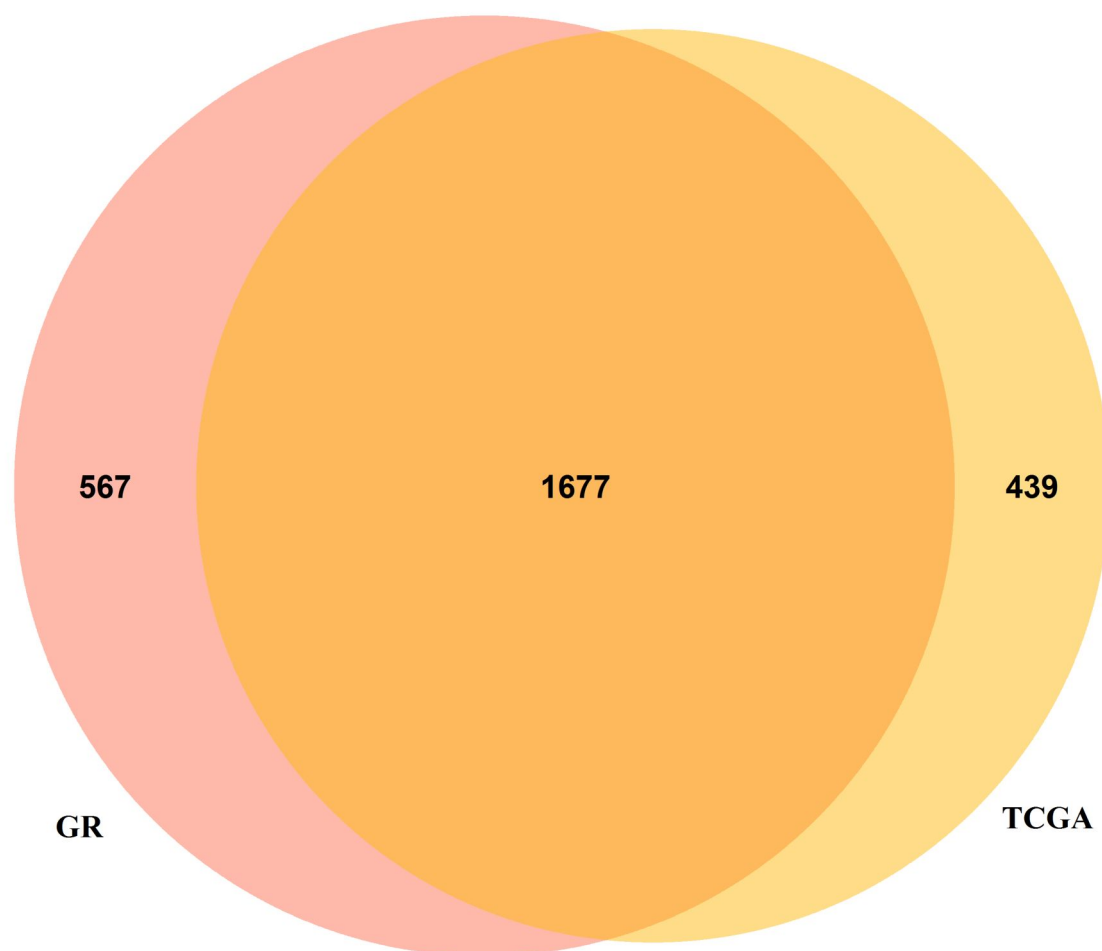

Supplement: S2 Fig — (PDF) [file pmed.1002201.s002.pdf]

# ESR1

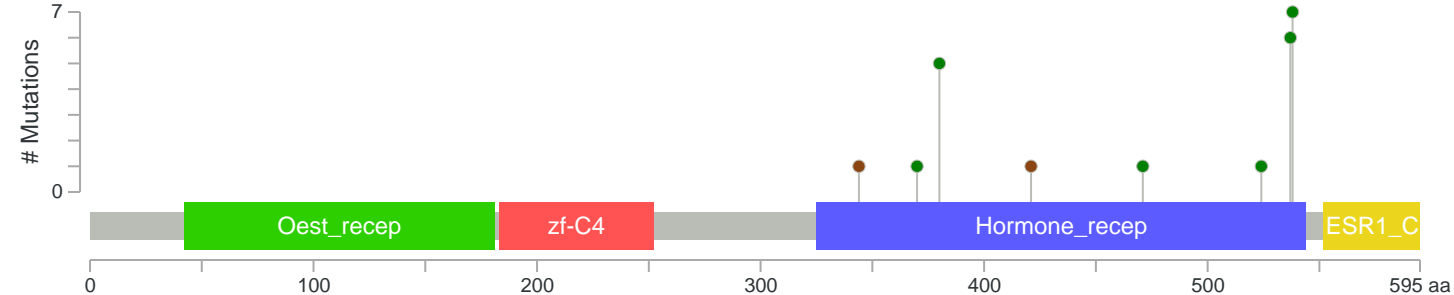

Supplement: S3 Fig — Green dots represent missense mutations, while brown dots represent indels. (PDF) [file pmed.1002201.s003.pdf]

RB1

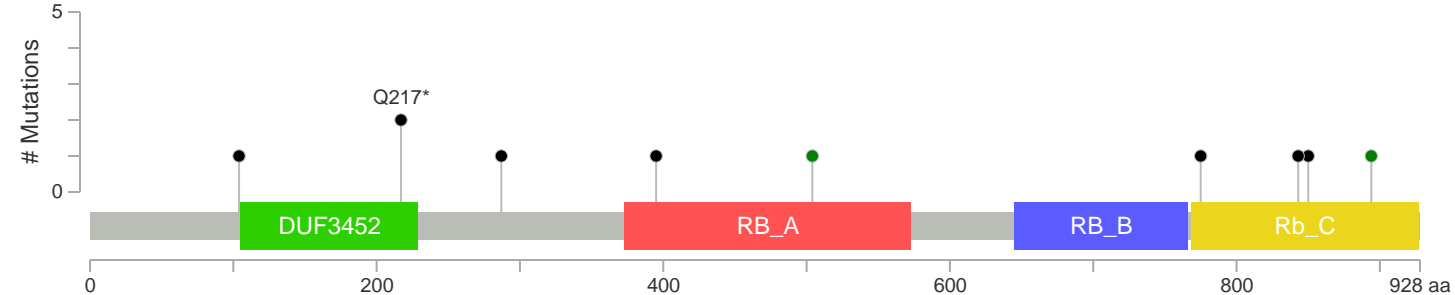

Supplement: S4 Fig — Green dots represent missense mutations, while black dots represent truncating mutations. (PDF) [file pmed.1002201.s004.pdf]

### Signature 1 (de novo)

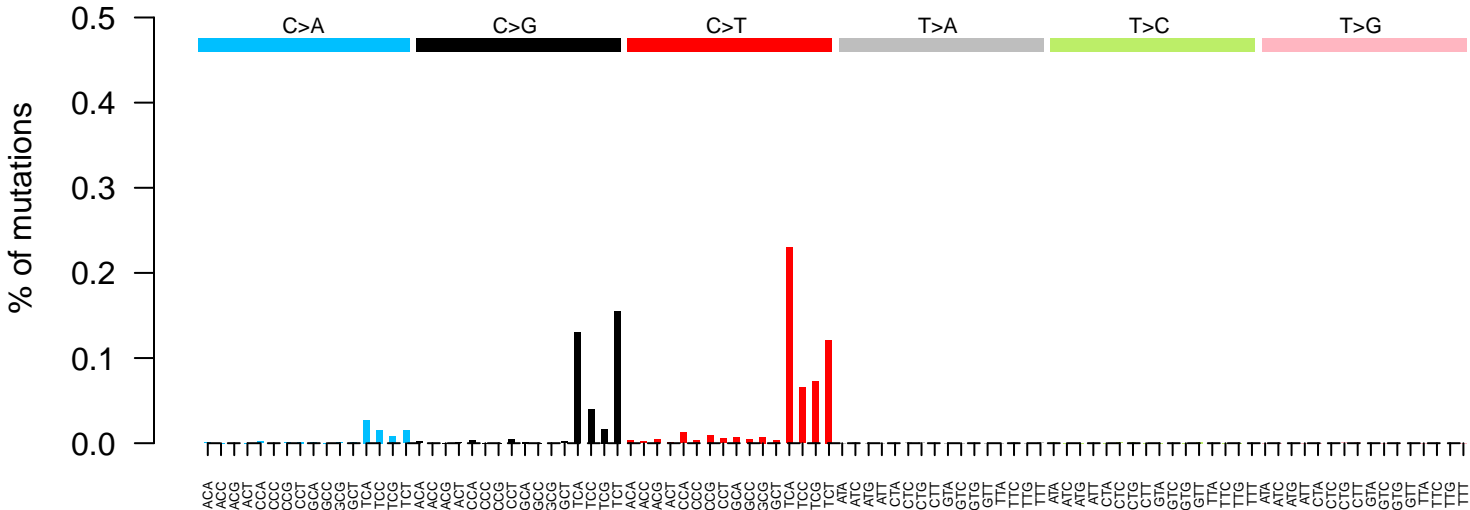

## Signature 2 (de novo)

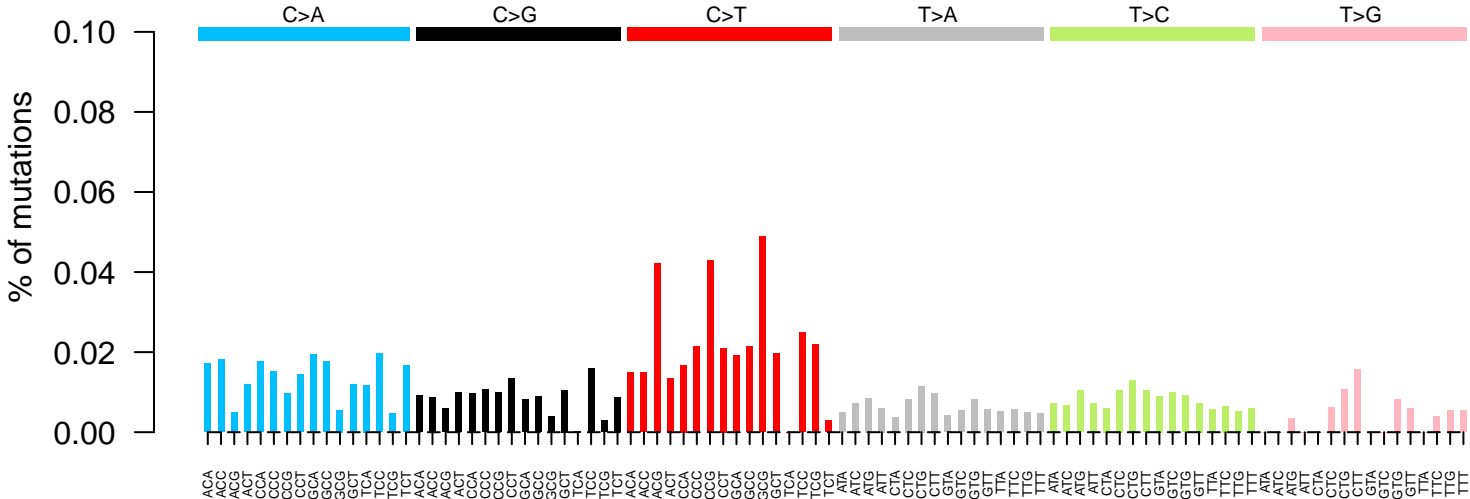

Supplement: S5 Fig — (PDF) [file pmed.1002201.s005.pdf]

**Signature 1 (de novo)**

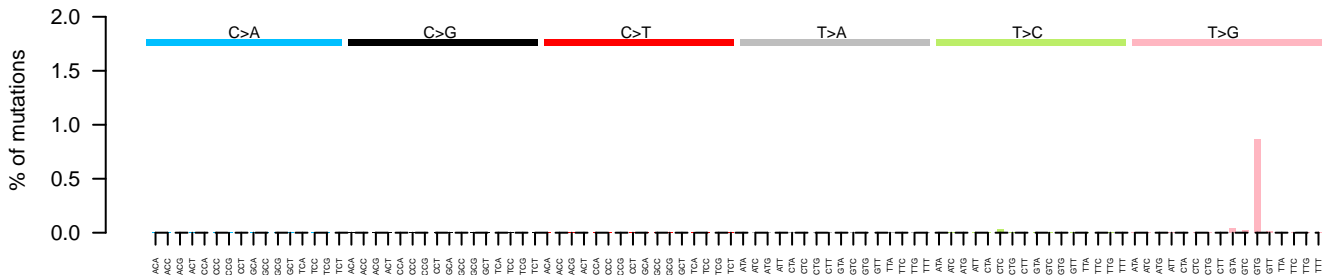

### Signature 2 (de novo)

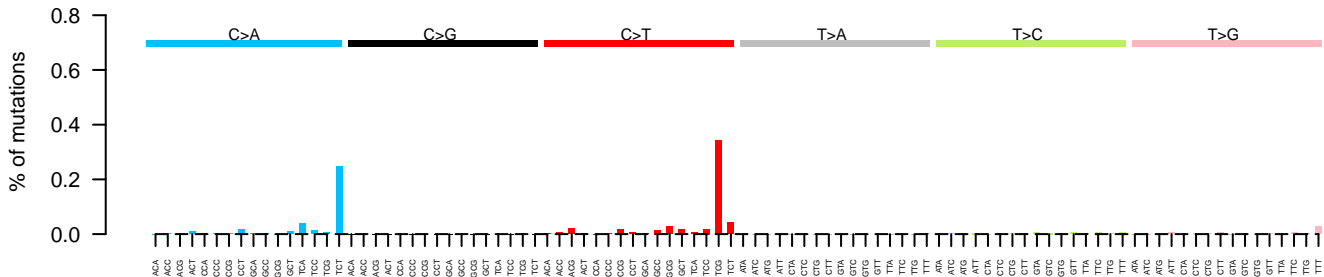

**Signature 3 (de novo)**

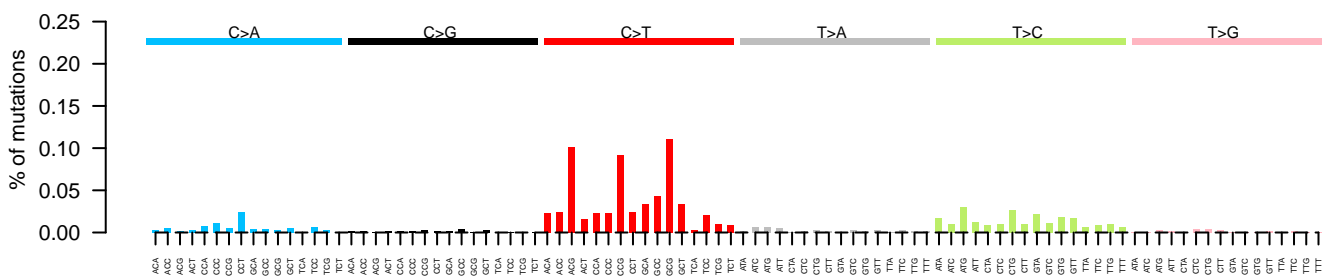

### Signature 4 (de novo)

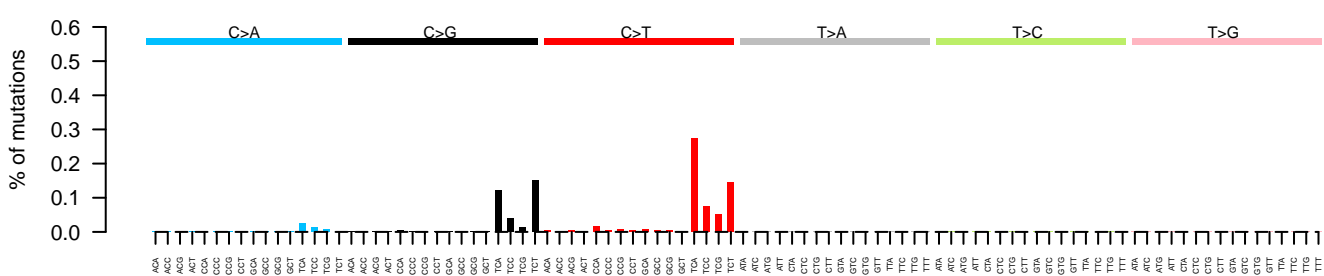

### Signature 5 (de novo)

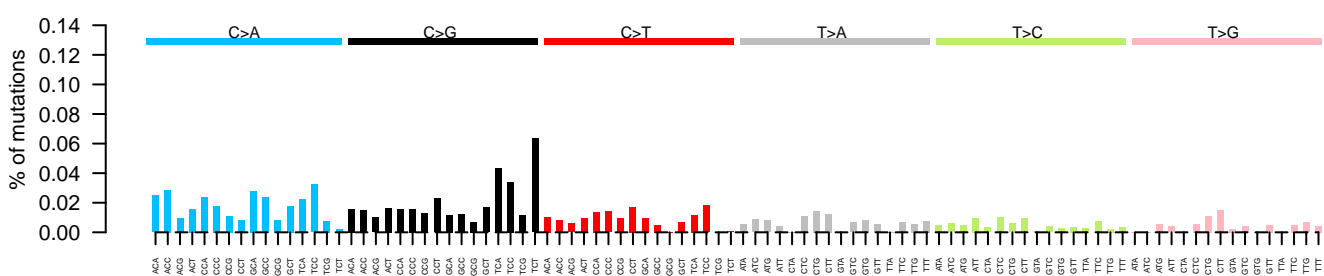

Supplement: S6 Fig — (PDF) [file pmed.1002201.s006.pdf]

## TN patient

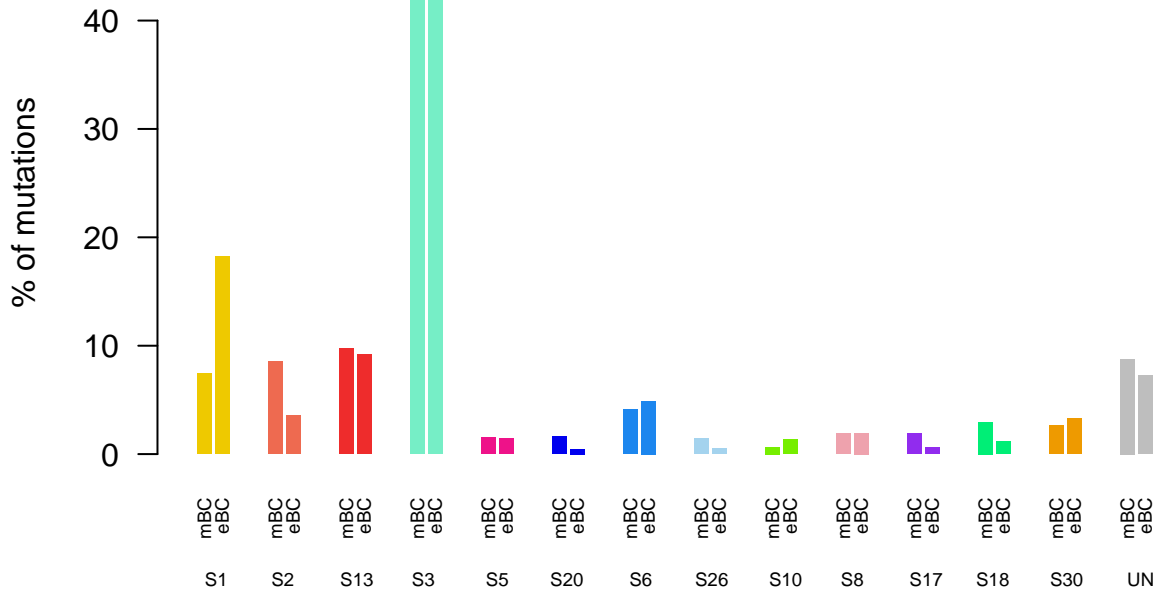

Supplement: S7 Fig — (PDF) [file pmed.1002201.s007.pdf]

## HER2+ patient

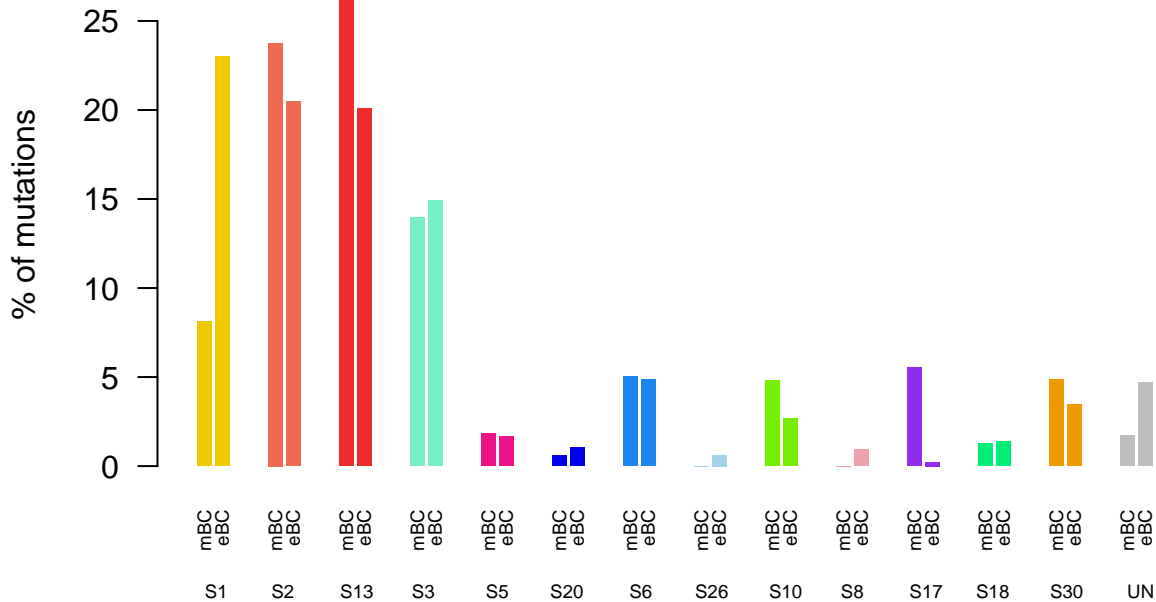

Supplement: S8 Fig — (PDF) [file pmed.1002201.s008.pdf]

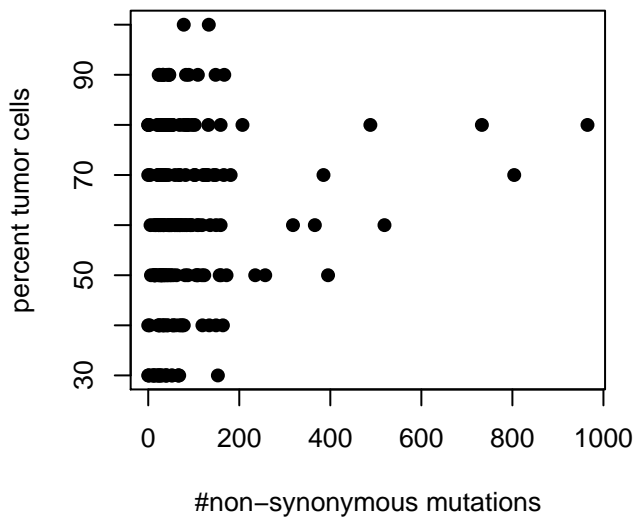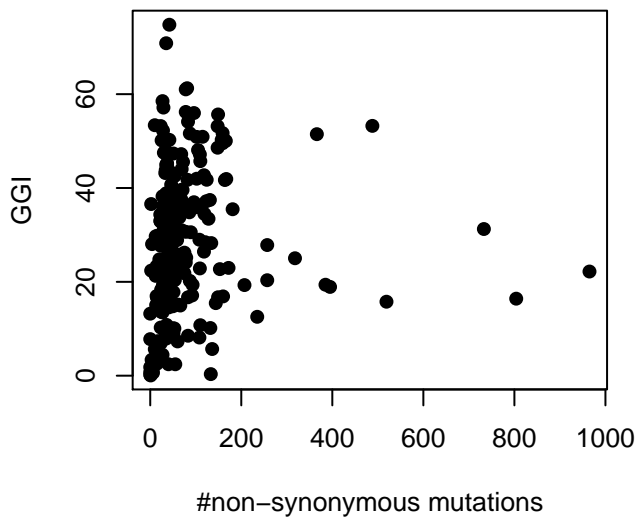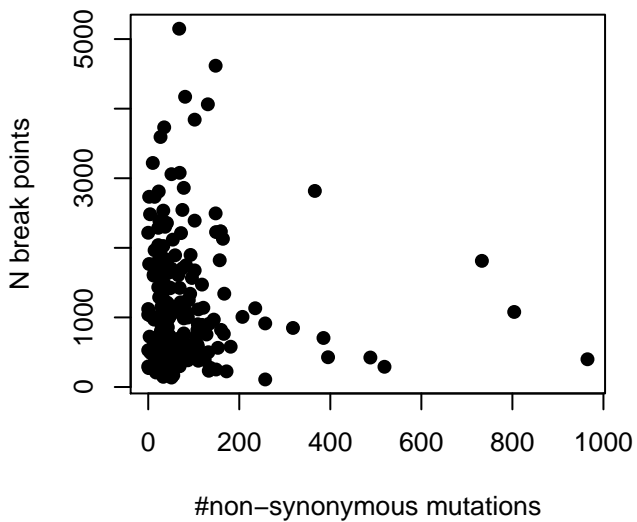

Supplement: S9 Fig — (PDF) [file pmed.1002201.s009.pdf]
