# Supplementary material for: Mutational Profile of Metastatic Breast Cancers: A Retrospective Analysis
Source: PLoS Med. 2016 Dec 27;13(12):e1002201. doi: 10.1371/journal.pmed.1002201 (PMC5189935; doi:10.1371/journal.pmed.1002201)
Supplement: S7 Table — (DOCX) [file pmed.1002201.s016.docx]

S7 Table : Univariate analysis

| Evt / N S(6mo) HR 95%CI p |
| --- |
| Age at inclusion (cl) : p = 0.4162  <=50 y.o. 55 / 91 90.84% 1.00  >50 y.o. 71 / 125 82.92% 0.86 [ 0.61; 1.23]    Nb of Met sites (cl) : p = 0.0026  1-2 65 / 123 89.92% 1.00  >2 61 / 92 81.45% 1.71 [ 1.20; 2.43]    Diag Met - Inclusion delay(cl) : p = 0.6661  <12 mo 67 / 113 82.84% 1.00  12-24 mo 14 / 31 86.31% 0.78 [ 0.44; 1.39]  >24 mo 41 / 65 90.77% 1.01 [ 0.69; 1.49]    Prior Chemotherapy : p = 0.0016  No 3 / 16 100.00% 1.00  Yes 123 / 200 85.16% 5.24 [ 1.66;16.50]  IHC : p = 0.0001  HR+/HER2- 78 / 143 91.41% 1.00  HR-/HER2- 37 / 51 67.16% 2.11 [ 1.42; 3.12]  HER2+ 6 / 14 92.86% 0.56 [ 0.24; 1.29]  Hormonal Treatment (Neo / Adj or Met) : p = 0.0186  No 50 / 73 75.67% 1.00  Yes 76 / 143 91.47% 0.65 [ 0.46; 0.93]    Liver Met: p = 0.0217  No 49 / 92 87.78% 1.00  Yes 77 / 123 85.05% 1.52 [ 1.06; 2.18]    **At least one gene mutated ((ESR1, FSIP2 , ...)): p = 0.0237**  **No 76 / 140 87.54% 1.00**  **Yes 50 / 76 83.72% 1.51 [ 1.05; 2.16]** |
|  |
