# Supplementary material for: Mutational Profile of Metastatic Breast Cancers: A Retrospective Analysis
Source: PLoS Med. 2016 Dec 27;13(12):e1002201. doi: 10.1371/journal.pmed.1002201 (PMC5189935; doi:10.1371/journal.pmed.1002201)
Supplement: S8 Table — (DOCX) [file pmed.1002201.s017.docx]

S8 Table : Mutlivariate analysis

| HR 95%CI p |
| --- |
| N met sites (cl) :  1-2 1.000 (base)  >2 1.428 [0.985 ; 2.072] 0.060  IHC  HR+/HER2- 1.000 (base)  HR-/HER2- 1.912 [1.158; 3.156] 0.011  HER2+ 0.558 [1.368; 0.202] 0.228  Prior Chemotherapy :  No 1.000 (base)  Yes 6.699 [2.055 ; 21.839] 0.002  Hormonal Treatment. (Neo / Adj or Met)  No 1.000 (base)  Yes 0.446 [0.271 ; 0.732] 0.001  Liver Met  No 1.000 (base)  Yes 2.143 [1.398; 3.285] <0.001  **At least one gene mutated ((ESR1, FSIP2 , ...))**  **No 1.000 (base)**  **Yes 1.966 [1.339; 2.888] 0.001** |
