# Supplementary material for: Mutational Profile of Metastatic Breast Cancers: A Retrospective Analysis
Source: PLoS Med. 2016 Dec 27;13(12):e1002201. doi: 10.1371/journal.pmed.1002201 (PMC5189935; doi:10.1371/journal.pmed.1002201)
Supplement: S1 Text — (DOCX) [file pmed.1002201.s020.docx]

Supplementary text for

**Mutational profile of metastatic breast cancers: a retrospective analysis**

Celine Lefebvre^1^, Thomas Bachelot^2^, Thomas Filleron^3^, Marion Pedrero^1^, Mario Campone^4^, Jean-Charles Soria^1,5,6,7^, Christophe Massard^7^, Christelle Lévy^8^, Monica Arnedos^5^, Magali Lacroix-Triki^1^, Julie Garrabey^9^, Yannick Boursin^10^, Marc Deloger^10^, Yu Fu^1^, Frédéric Commo^1^, Véronique Scott^1^, Ludovic Lacroix^1,11^, Maria Vittoria Dieci^12,13^, Maud Kamal^14^, Véronique Diéras^14^, Anthony Gonçalves^15^, Jean-Marc Ferrerro^16^, Gilles Romieu^17^, Laurence Vanlemmens^18^, Marie-Ange Mouret Reynier^19^, Jean-Christophe Théry^20^, Fanny Le Du^21^, Séverine Guiu^22^, Florence Dalenc^23^, Gilles Clapisson^24^, Hervé Bonnefoi^25^, Marta Jimenez^9^, Christophe Le Tourneau^14,26^, Fabrice André^1,5,6*^

**Detailed description of the clinical trials:**

The SAFIR01 trial is an observational study where the investigators performed high throughput molecular technologies to drive metastatic breast cancer patients into specific phase I/II trials. Key inclusion criteria included: men and women with histologically diagnosed breast cancer and with metastatic relapse or stage IV breast cancer at diagnosis; the metastases are amenable to biopsy; patients are <70 years old with performance status of 0 or 1. There was no restriction regarding the number of previous chemotherapy or endocrine therapies. The period of inclusion went from June 2011 to July 2012. This study was conducted in accordance with the ethical standards of the responsible committee on human experimentation (institutional and national). The study was approved by an independent ethics committee (CPP Ile de France VII) and the French National Health Authorities (ANSM). The study was registered in ClinicalTrials.gov (NCT01414933) database. All patients gave written informed consent before participating in the trial

The SAFIR02 trial is an open label multicentric phase II randomized trial, using high throughput genome analysis as a therapeutic decision tool, which aims at comparing a targeted treatment administered according to the identified molecular anomalies of the tumor with maintenance chemotherapy or an immunotherapy with maintenance chemotherapy in patients with no actionable genomic alterations. Key inclusion criteria included: women or men with histologically proven breast cancer with metastatic relapse or progression or stage IV at diagnosis; No Her2 over-expression; Patients who are eligible for a first or a second line of chemotherapy in metastatic setting, or who are currently treated with a first or second line of chemotherapy with a maximum of 2 cycles at the time of biopsy. For patients with ER+ disease, relapse or progression occurred during endocrine therapy, whatever the line, or less than 12 months after the end of endocrine therapy in adjuvant context; WHO performance status of 0 or 1. The trial is ongoing with a period of inclusion started in March 2014. This study was conducted in accordance with the ethical standards of the responsible committee on human experimentation (institutional and national). The study was approved by an independent ethics committee (CPP Ile de France II) and the French National Health Authorities (ANSM). The study was registered in ClinicalTrials.gov (NCT02299999) database. All patients gave written informed consent before participating in the trial.

The SHIVA trial is a proof of concept randomized phase II trial which compares two treatment strategies for patients with refractory cancer. If a molecular abnormality is identified for which an approved targeted agent is available, patients are randomized between two arms: targeted therapy based on the molecular profile and conventional therapy based on investigator's choice. Key inclusion criteria included: patients with recurrent/metastatic solid tumor who failed or are not candidate for treatments usually proposed in first intentions and for whom a prospective clinical trial has been indicated in a tumor board, ECOG performance status of 0 or 1. The period of inclusion went from October 2012 to July 2014. The study was approved by an independent ethics committee (CPP) and the French National Health Authorities (ANSM). The study was registered in ClinicalTrials.gov (NCT01771458) database. All patients gave written informed consent before participating in the trial.


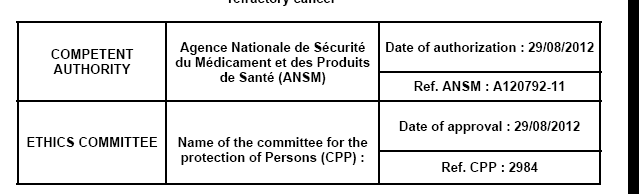


The MOSCATO trial is a prospective molecular screening trial that uses high-throughput molecular analysis to select molecular targeted therapies to patients with metastatic cancer and actionable molecular alterations. Based on their genomic profiles, patients were treated with the most relevant targeted therapy available through early clinical trials or marketed molecular targeted agents. Key inclusion criteria included: uncurable solid tumors of stage IV with local relapse or metastasis; performance status of 0 or 1 or Lansky play scale >= 70%; Minimum one treatment line, no limit in the prior number of treatment line. The period of inclusion went from December 2011 to March 2016. The study was approved by an independent ethics committee and the French National Health Authorities (ANSM). The study was registered in ClinicalTrials.gov (NCT01566019) database. All patients gave written informed consent before participating in the trial.

**De novo mutational signature analysis**

In order to identify the mutational signatures operative in metastatic breast cancer, we performed de novo mutational signature analysis with the Matlab Welcome Trust Sanger Institute’s algorithm [25]. We first analyzed mutational profiles of 213 metastatic tumors with at least one mutation that revealed 2 signatures (Supplementary Figure 5). Signature de novo 1 related to the COSMIC signatures 2 and 13 (APOBEC-related mutational process) while signature de novo 2 related to both COSMIC signature 1 (spontaneous deamination of 5-methylcytosine) and COSMIC signature 3 (failure of DNA double-strand break-repair by homologous recombination). Next, we analyzed the metastatic tumor mutation profiles together with the TCGA primary tumor mutation profiles in order to gain in sensitivity in mutational process identification. This analysis revealed 5 signatures operative in metastatic and primary tumors (Supplementary Table 9 and Supplementary Figure 6).
